# Distributive differences of P2Xs between the forelimb and hind limb of adjuvant arthritis rats and intervention by Notopterygh rhizoma et radix

**DOI:** 10.1080/13880209.2018.1561730

**Published:** 2019-02-06

**Authors:** Yinghao Wang, Zhihuang Chen, Chen Liu, Xuehua Lu, Ce Yang, Songping Qiu

**Affiliations:** a College of Pharmacy, Fujian University of Traditional Chinese Medicine, Fuzhou, PR China;; b College of Chemical and Material Science Engineering, Kaili University, Guizhou, PR China;; c Fujian Medical Science Research Institute, Fuzhou, PR China

**Keywords:** P2X receptor subtypes, rheumatoid arthritis, arthromyodynia, articular cavity, paw edema

## Abstract

**Context:***Notopterygium incisum* Ting ex H. T. Chang (Umbelliferae) (NI) specializes in treatment of upper limb rheumatoid arthritis (RA), but the exact mechanism is unclear. P2Xs are useful targets for inflammatory pain therapy. It led us to hypothesize that NI may preferentially act on particular P2Xs and these receptors may be unevenly distributed in the upper/lower limb.

**Objective:** To investigate P2Xs distribution in the upper/lower limb and NI's targets in upper limb RA.

**Materials and methods:** The SD rats were randomized into 11 groups of 10 animals each. Eight experimental groups were established by the injection of 0.1 mL FCA into the plantar surface of rat paw. Three control groups suffered the same volume of saline. The articular cavities were then taken on the seventh day to detect P2Xs expression. NI (3 g/kg) and prednisone (10 mg/kg) were respectively given by oral gavage once daily for 14 d. The swelling degree and P2Xs were evaluated individually.

**Results:** In normal rats, the expressions of P2X_3_ and P2X_6_ in forelimb were markedly higher than that of in hind limb (*P* < 0.05). After induced by FCA, P2X_1_, P2X_3_, P2X_4_, P2X_5_ and P2X_7_ were increased significantly (*P* < 0.01). The biggest difference was P2X_3_. In NI treatment rats, swelling degree of the 7th/14th day in forelimb was 68.24%/38.89%, whereas that of in hind limb was 88.72%/79.92%. P2X_3_ mRNA and protein expression was significantly reduced as contrasted with the control group (*P* < 0.05).

**Conclusions:** P2X_3_ receptor was predominantly expressed in the forelimb RA rat. NI relieved the FCA-induced RA by inhibiting upper limb’s P2X_3_ receptor.

## Introduction

Purinoceptors P2Xs are a family of ligand-gated ion channels, activated by extracellular ATP (Dunn et al. 2001). Seven distinct genes encode P2X receptors (P2X_1-7_). They are homo- (e.g., P2X_1_ in smooth muscle cells as well as P2X_2_ to P2X_4_ and P2X_7_) or heteropolymers (e.g., P2X_2_-P2X_3_ and P2X_1_-P2X_5_) (Thiriet 2012). Studies have shown that they are prevalently expressed in parasympathetic, sympathetic, spinal cord, pelvic and myenteric neurons, digit and associated vasculature, as well as sensory and adrenomedullary chromaffin cells (Stanfa et al. 2000; Dunn et al. 2001; Zamboulis et al. 2013), and they play an important role in physiological and pathological conditions (Cockayne et al. 2000; Stanfa et al. 2000; Eltzschig et al. 2012; Zamboulis et al. 2013; Kurashima et al. 2015). Under physiological condition, the binding of extracellular ATP to trimeric P2X receptors triggers the opening of a transmembrane pore, allowing Ca^2+^, Na^+^, and K^+^ to change their membrane voltage, reduces electrochemical gradients, and activates intracellular signaling cascades (Thiriet 2012). Under injurious condition, inflammation causes a double-triple enhancement in ATP-activated currents, alter the P2X receptors’ voltage dependence, and increase the P2X receptors’ expression. Current research shows P2X receptors up-regulation can lead to abnormal pain responses concerned with inflammatory injuries. (Xu and Huang 2002; Li et al. 2018). It indicates that P2X receptors are one of the useful targets in treatment of inflammatory pain (Souslova et al. 2000; Xu and Huang 2002; Fiebich et al. 2014; Teixeira et al. 2017). Inflammatory pain is common in rheumatoid arthritis (RA). RA is a common chronic autoimmune disease noted for inflammation of joint synovium and destruction of articular cartilage (Breedveld et al. 2006). Its etiology is not yet clear. Though most traditional anti-rheumatic drug therapies have been confirmed to reduce joint destruction, optimal therapeutic drug does not stop developing (Breedveld et al. 2006; Smolen et al. 2010). Traditional Chinese Medicine (TCM) has long treated RA and categorizes RA as arthromyodynia. The most commonly used Chinese medicine is *Notopterygium incisum* Ting ex H. T. Chang (Umbelliferae) (Qiang-Huo, NI), which specializes in the treatment of arthromyodynia in upper limb. NI is widespread throughout western China. As a type of TCM, it has been used to treat cold, pain and upper limb arthromyodynia (Editorial Committee of Chinese Materia Medica 2000).

In current clinical practice, NI has been widely used in RA, especially in that of upper limb (Li 2012; Zhang 1999). In our previous studies, we had also found that it could significantly improve the forelimb pain threshold of mechanical stress stimulation in adjuvant arthritis (AA) rat model (Liu et al. 2015). Why is it only good at treating arthromyodynia in the upper limb? What is its direct target on upper limb? The mechanism is not yet clear. In light of receptors P2Xs' roles in the transmission signals of inflammatory pain and different distribution in organs (Zhang and Zhao 2005; Muller 2010; Zamboulis et al. 2013; Franceschini and Adinolfi 2014), it led us to hypothesize that P2X receptor subtypes may be unevenly distributed in the upper and lower limbs for RA, and NI may act on the pain by affinity with the differential subtypes.

This research investigates P2X receptor subtypes distribution in the upper and lower limbs and NI's targets in upper limb RA. The specific objectives of the present study included: (1) Differential expression of P2Xs in forelimb/hind limb’s articular cavities of rats. (2) Differential expression of P2Xs in forelimb/hind limb’s articular cavities of AA rats. (3) The therapeutic effect of NI on AA model. (4) RT-PCR and Western blot detection of differential subtype expression in forelimb's articular cavities after treatment by NI.

## Materials and methods

### Chemicals and reagents

Freund's complete adjuvant (FCA) was obtained from Sigma (St. Louis, MO). Gene-specific primers were supplied by Sangon Biotech Co., Ltd. (Shanghai, China). Trizol reagent, Taq polymerase, DNA Marker, Gelstain Nucleic Acid Gel Stain, TransScript One-Step gDNA Removal and cDNA Synthesis SuperMix were purchased from TransGen Biotech (Beijing, China). Bicinchoninic acid (BCA) protein determination and enhanced chemiluminescence (ECL) plus were obtained from Vazyme Biotech Co., Ltd. (Nanjing, China). All the other chemicals were provided from common commercial sources and were at least of analytical grade.

### Animal preparation

All the experimental procedures complied with the National Institutes of Health Guide for the Care and Use of Laboratory Animals. The animal protocol was approved by the Ethics Committee of Fujian University of TCM (Fuzhou, China). One hundred and ten healthy male Sprague-Dawley (SD) rats (180–220 g) were provided by SLRC laboratory animal Co., Ltd. (Shanghai, China). The SD rats were randomly divided into control and experimental groups (control group, 30 rats; experimental group, 80 rats) and housed in controlled environments at controlled temperature (25 ± 1 °C) under a 12 h dark-light cycle. Commercial rat feedstuff and distilled water were available *ad libitum*.

### FCA-induced rat model of rheumatoid arthritis and tissue preparation

After acclimatizing for 1 week, the RA model of experimental groups (20 rats) was established by the injection of 0.1 mL FCA into the plantar surface of the rat’s paw. The injection sites were right front paw/right hind paw to differentiate forelimb/hind limb of AA model (Liu et al. 2015). The control group containing 10 rats suffered the same volume of saline. On the seventh day after successfully induced by FCA, the anesthetized and bloodletting rats were prostrate on the sterile table. Then articular cavities were taken respectively. The marked articular cavities were promptly frozen by liquid nitrogen and then stored at ˗80 °C until RT-PCR experiment.

### Effect of NI on AA rats

The extraction process of NI was identical to those detailed earlier (Xie et al. 2014; Liu et al. 2015). The extract mainly contained coumarin compounds, including isoimperatorin, columbianetin, bergapten, and so on (Xie et al. 2014). Eighty animals were randomized into eight groups of 10 rats each. Four groups in forelimb’s experiment were examined: control group, model group, prednisone treatment group (positive group, 10 mg/kg), and NI treatment group (NI group, 3 g/kg). There were the same groups and dosages in the hind limb's experiment. The identical modeling method described above was applied. After successfully induced by FCA, treatment groups were given corresponding medicines by oral gavage once daily for 14 d. On the 7th and 14th day, the rat paw edema was determined respectively by Volume Meter (Anhui Zhenghua Biological Instrument Equipment Co. LTD., China). The swelling degree was calculated according to Equation (1). After the last gavage, articular cavities were taken as before and stored at ˗80 °C for RT-PCR and Western blot detection.

(1) $$\%\text{ swelling degree}=\left({\text{V}}_{\text{treatment group}}-{\text{V}}_{\text{control group}}\right)/\left({\text{V}}_{\text{model group}}-{\text{V}}_{\text{control group}}\right)\times \text{1}00\%$$

### Analysis of P2Xs mRNA levels

#### Total RNA extraction and quantitation

Total RNA isolation was carried out using Trizol Reagent. Its purity and concentration was determined by a NanoDrop 2000 C Spectrophotometer (Thermo Scientific, Waltham, MA). Its completeness and contamination was identified by 2% agarose gel electrophoresis. The results showed that the ratios of OD_260_/OD_280_ were all between 1.8 and 2.0, indicated that RNA purity was satisfactory. Two clear bands of 28 S and 18 S suggested that RNA was complete. Thus, it complied with subsequent experimental requirements.

#### RT-PCR analysis of P2Xs mRNA levels

RNA (1 µg) was reverse-transcribed in accordance with the supplier’s instructions using SuperScript First-Strand Synthesis System. Reaction conditions were as follows: incubation at 42 °C for 30 min, heating at 85 °C for 5 min. The achieved cDNA was applied to determine the mRNA amount of P2X_1_, P2X_2_, P2X_3_, P2X_4_, P2X_5_, P2X_6_ and P2X_7_ by PCR. GAPDH, a housekeeping gene, served as an internal reference. The primer sequences used for amplification of P2X_1_, P2X_2_, P2X_3_, P2X_4_, P2X_5_, P2X_6_, P2X_7_ and GAPDH transcripts were as follows: P2X_1_ forward 5′ GTG CCC AGT CTT CAA CCT TGG CTA TG 3′ and reverse 5′ AAG AGG TGA CGA CGG TTT GTC CCA TT 3′; P2X_2_ forward 5′ CAT TCA GAC TGT ATT GC 3′ and reverse 5′ TGG ATG CTG TTC TTG ATG AGG AT 3′; P2X_3_ forward 5′ TGG CGT TCT GGG TAT TAA GAT CGG 3′ and reverse 5′ CAG TGG CCT GGT CAC TGG CGA 3′; P2X_4_ forward 5′ GAG GCA TCA TGG GTA TCC AGA TCA AG 3′ and reverse 5′ GAG CGG GGT GGA AAT GTA ACT TTA G 3′; P2X_5_ forward 5′ CAT CTT CCG ACT GGG GTC TAT TGT 3′ and reverse 5′ CTC AAC ATT GGC ATC CTC CTT CTG 3′; P2X_6_ forward 5′ ATG TGG CTG ACT TCG TGA G 3′ and reverse 5′ AGG TAT CTA AGG CAT TGG TTC TG 3′; P2X_7_ forward 5′ GTG CCA TTC TGA CCA GGG TTG TAT AAA 3′ and reverse 5′ GCC ACC TCT GTA AAG TTC TCT CCG ATT 3′; GAPDH forward 5′ ACG GCA AGT TCA ACG GCA CAG 3′ and reverse 5′ GAA GAC GCC AGT AGA CTC CAC GAC 3′. Thermal cycling conditions of P2X_1_, P2X_2_ and P2X_6_ were applied: initial denaturation at 94 °C for 5 min, followed by 94 °C (denaturation for 30 s), 58 °C (annealing for 45 s) and 72 °C (extension for 20 s) for the overall number of 35 cycles, terminal extension at 72 °C for 4 min. Thermal cycling conditions of P2X_3_, P2X_4_, P2X_5_ and P2X_7_ were applied: initial denaturation at 94 °C for 5 min, followed by 94 °C (denaturation for 30 s), 58 °C (annealing for 60 s) and 72 °C (extension for 90 s) for the overall number of 35 cycles, terminal extension at 72 °C for 8 min. Amplification PCR products (2 μL) were separated by 1.5% agarose gel electrophoresis, visualized by ethidium bromide staining, and analyzed by gel automatic imaging analysis system (BIO-RAD, Hercules, CA). The mRNA level was expressed as the ratio to GAPDH.

### Western blot analysis of P2X_3_ protein expression

Total proteins of articular cavities were extracted by radioimmunoprecipitation assay lysis buffer. The determination of protein concentration was set up using the BCA method. Protein (50 µg) per lane was fractionated by standard gel, transferred onto a 0.45 µm PVDF membrane (EMD Millipore, Billerica, MA), and blocked with 5% milk dissolved in PBST for 2 h. The membranes were incubated overnight at 4 °C with primary antibody P2X_3_ (1:500 in 5% milk in PBST) and then with secondary antibody (1:10,000 in 5% milk in PBST) at room temperature for 1 h followed by ECL detection. P2X_3_ protein expression was presented as the ratio to GAPDH.

### Data analysis

The experimental data were presented as means ± standard deviations. The statistical analyses were carried out using one-way analysis of variance (ANOVA) followed by LSD *post hoc* tests. *P*-values < 0.05 were accepted as statistically significant.

## Results

### Differential expression of P2Xs in forelimb/hind limb's articular cavities of control groups

**Figure 1.**
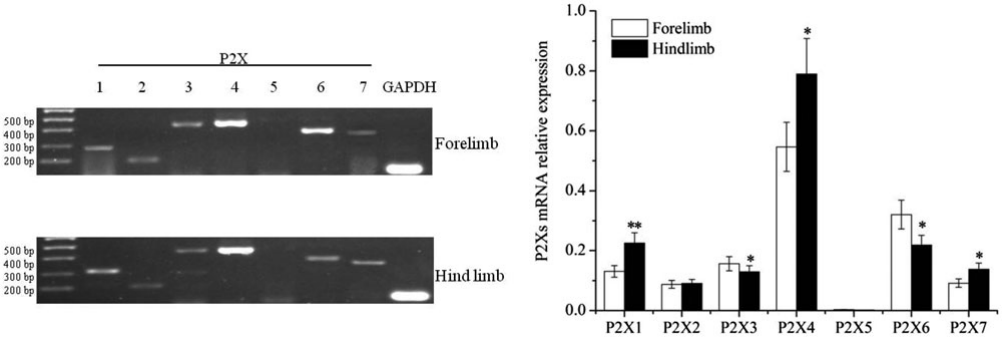
Analysis of P2X receptor subtypes expression in forelimb/hind limb's articular cavities of control groups. *P < 0.05, **P < 0.01, comparison between groups.

P2X receptor subtypes expression of forelimb's articular cavities in control groups were shown in Figure 1. It could be seen that the electrophoresis band of P2X_5_ receptor was ambiguous. The rest of the subtypes were all stable expressions. The types of P2X receptor subtypes expression in hind limb's articular cavities were similar to that of the forelimb's articular cavities. But the expression quantities were different in two groups, except that of P2X_2_ and P2X_5_. The expression quantities of P2X_1_, P2X_4_ and P2X_7_ in hind limb were markedly higher than that in forelimb (*P* < 0.05). However, P2X_3_ and P2X_6_ were contrary (see Figure 1). It revealed that P2X receptor subtypes expression were different in forelimb and hind limb's articular cavities of rat.

### Differential expression of P2Xs in forelimb/hind limb's articular cavities of model groups

#### Expression in forelimb's articular cavities

**Figure 2.**
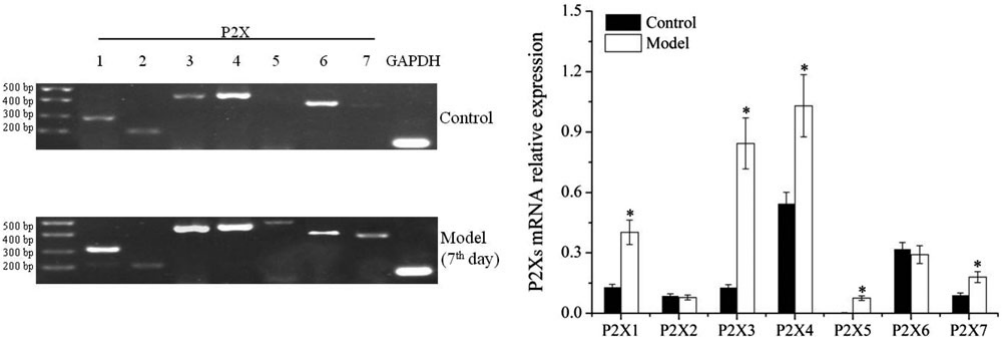
Analysis of P2X receptor subtypes expression in forelimb's articular cavities of model group. * *P* < 0.01, versus control.

After induced by FCA, P2X_1–7_ receptor subtypes in forelimb's articular cavities were stable expression (see Figure 2). The expression quantities of P2X_1_, P2X_3_, P2X_4_, P2X_5_ and P2X_7_ receptors increased greatly in model groups as compared with that in control group (*P* < 0.01). There were not obvious differences in P2X_2_ and P2X_6_ receptors. It indicated that the expression of P2X_1_, P2X_3_, P2X_4_, P2X_5_ and P2X_7_ receptors were increased significantly in forelimb's articular cavities of AA model.

#### Expression in hind limb's articular cavities

**Figure 3.**
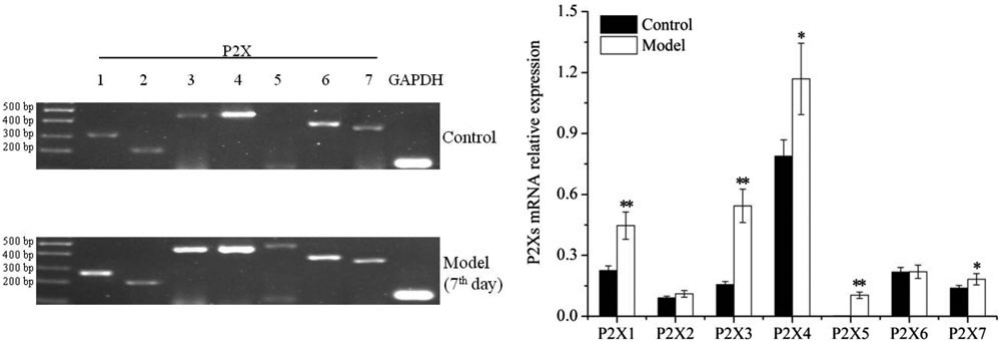
Analysis of P2X receptor subtypes expression in hind limb's articular cavities of model group. *P < 0.05, **P < 0.01, versus control.

The results of hind limb's articular cavities were similar to forelimb's articular cavities (see Figure 3). It implied that the expression of P2X_1_, P2X_3_, P2X_4_, P2X_5_ and P2X_7_ receptors in hind limb's articular cavities were increased remarkably after induced by FCA.

#### Differential expression in forelimb and hind limb's articular cavities

**Figure 4.**
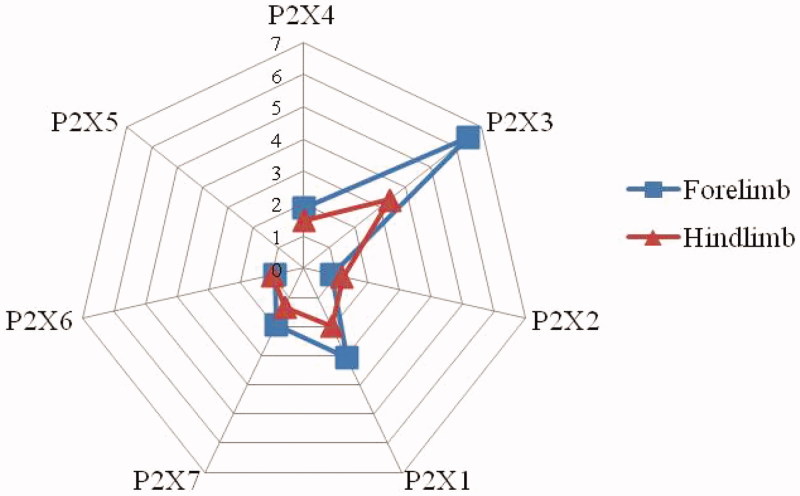
Differential expression of P2Xs in forelimb and hind limb's articular cavities. The value, representing a multiplier of P2X receptor subtypes expression, was equal to the expression of model limb over control limb.

As above, the expression of P2X_1_, P2X_3_, P2X_4_, P2X_5_ and P2X_7_ receptors increased remarkably after successfully induced by FCA, regardless of forelimb or hind limb. However, the degree of increase was different. The results are shown in Figure 4. The multiplier of P2X_1_, P2X_4_ or P2X_7_ receptor expression on forelimb was close to that of on hind limb. But the multiplier of P2X_3_ receptor expression on forelimb was 6.49, whereas that on hind limb was 3.46. P2X_3_ receptor expression on forelimb was considerably larger than that of on hind limb. The multiple of P2X_5_ receptor was not calculated for it was almost no expression in the control group, but the expression values in forelimb/hind limb of model groups were approximate each other. Collectively, these data indicated that the distribution of P2Xs was different in forelimb and hind limb of AA rats. The biggest difference was P2X_3_ receptor expression. It was significant increase in forelimb's experiment.

### The therapeutic effect of NI on AA model

#### Effect on forelimb of AA model

The therapeutic effect of NI on forelimb *in vivo* was evaluated by measuring rat paw edema. Data in Table 1 show that rat paw edema was very evident after model establishment (*P* < 0.01). As compared with the model group, rat paw edema in the treatment groups was significantly reduced regardless of 7 or 14 d (*P* < 0.05, *P* < 0.01). The results show that NI could significantly inhibit forelimb swelling of AA model.

**Table 1.** Change of rat paw edema in forelimb's experiment ($\bar{X}\pm S$, *n* = 10).

Groups	Before model establishment (mL)	After model establishment (mL)	7th day after treatment (mL)	14th day after treatment (mL)
Control group	0.53 ± 0.028	0.58 ± 0.039**	0.64 ± 0.059**	0.75 ± 0.098**
Model group	0.52 ± 0.035	1.27 ± 0.12	1.49 ± 0.23	1.65 ± 0.12
Positive group	0.55 ± 0.036	1.29 ± 0.13	1.10 ± 0.094**	1.10 ± 0.16**
NI group	0.54 ± 0.024	1.31 ± 0.11	1.22 ± 0.15*	1.10 ± 0.16**

* Significantly different from model group, *P* < 0.05;

** Significantly different from model group, *P* < 0.01.

#### Effect on hind limb of AA model

The *in vivo* effect of NI on hind limb was determined using rat paw edema. As shown in Table 2, swelling was significant in model group contrasting with control group (*P* < 0.01). After treatment, rat paw edema was alleviated in the treatment groups. But the difference in NI group was not significant from a statistical standpoint. It suggested that NI had less effect on hind limb swelling of AA model.

**Table 2.** Change of rat paw edema in hind limb's experiment ($\bar{X}\pm S$, *n* = 10).

Groups	Before model establishment (mL)	After model establishment (mL)	7th day after treatment (mL)	14th day after treatment (mL)
Control group	1.40 ± 0.11	1.53 ± 0.098**	1.52 ± 0.079**	1.72 ± 0.12**
Model group	1.47 ± 0.097	3.46 ± 0.75	4.09 ± 0.24	4.11 ± 0.13
Positive group	1.38 ± 0.092	3.21 ± 0.66	3.43 ± 0.31*	3.21 ± 0.23*
NI group	1.41 ± 0.056	3.39 ± 0.61	3.80 ± 0.23	3.63 ± 0.30

*Significantly different from model group, *P* < 0.05;

**Significantly different from model group, *P* < 0.01.

#### Differential effect on forelimb and hind limb of AA model

**Figure 5.**
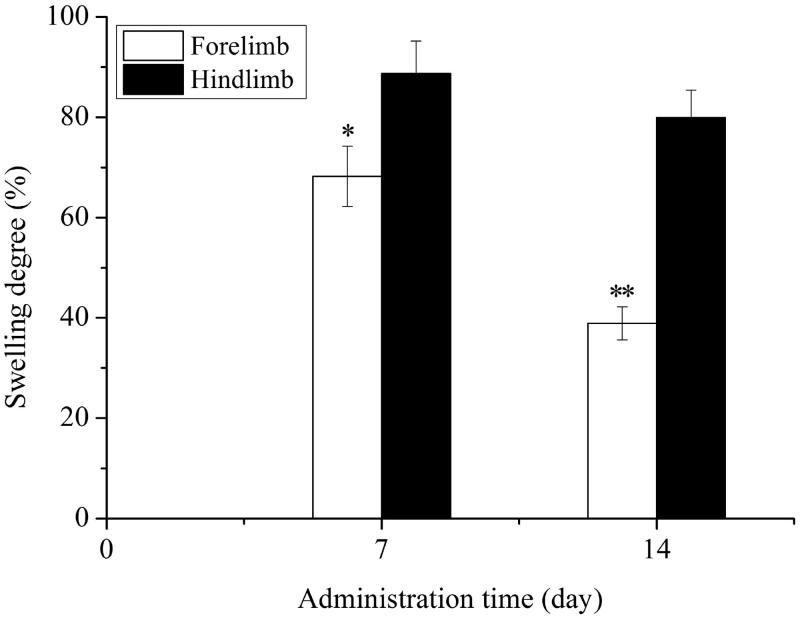
Swelling degree in forelimb and hind limb after treatment for 7 or 14 d by NI. *P < 0.05, **P < 0.01, versus hind limb.

As shown in Figure 5, swelling degree of the 7th day or 14th day in forelimb was 68.24% or 38.89%, whereas that in hind limb was 88.72% or 79.92%. There was remarkable difference between two groups (*P* < 0.05, *P* < 0.01). Collectively, these data suggested that the therapeutic effect of NI was mainly on forelimb of AA model.

### Expression of P2X_3_ after treatment by NI

#### Analysis of P2X_3_ mRNA level

**Figure 6.**
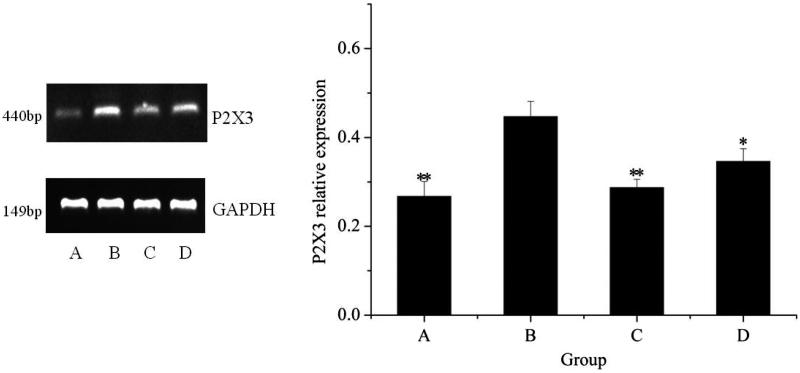
Analysis of P2X_3_ mRNA expression in forelimb's articular cavities. A, control group; B, model group; C, positive group; D, NI group. *P < 0.05, **P < 0.01, vs. controls.

To further reveal the mechanism of NI's therapeutic effect in forelimb's experiment, P2X_3_ mRNA and protein expression were respectively examined by RT-PCR and Western blot assay. It can be seen from Figure 6 that P2X_3_ mRNA expression was profoundly increased after model establishment (*P* < 0.01). But the treatment groups (NI group and positive group) significantly reduced P2X_3_ mRNA expression as contrasted with the control group (*P* < 0.05, *P* < 0.01).

#### Western-blot analysis of P2X_3_ protein expression

**Figure 7.**
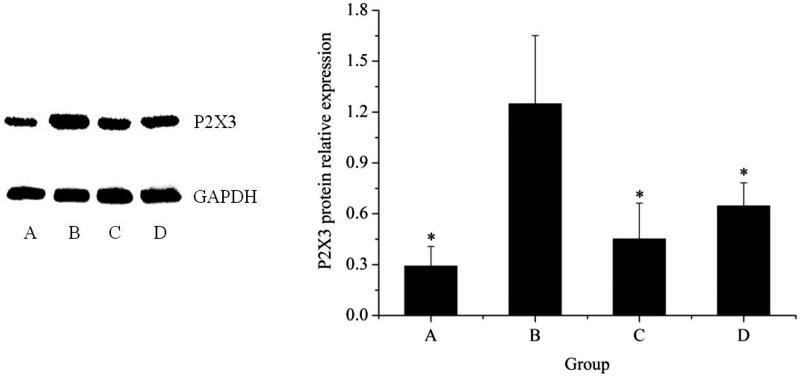
Analysis of P2X_3_ protein expression in forelimb's articular cavities. A, control group; B, model group; C, positive group; D, NI group. *P < 0.05, **P < 0.01, vs. controls.

Western blot analysis uncovered the similar effects on P2X_3_ protein expression as on mRNA level (*see*Figure 7). Taken together, it was demonstrated that NI down-regulated P2X_3_ expression in forelimb's articular cavities of AA model.

## Discussion

RA is a common autoimmune disease that has the characteristic of chronic, symmetrical, erosive and progressive destruction of peripheral joints (Chu et al. 2018). Therefore, it is necessary to establish a practical and reliable animal model to study the pathogenesis of RA and evaluate the efficacy of drugs. At present several animal models have been used extensively in studies of RA pathogenesis or drugs efficacy (Asquith et al. 2009). AA rat is the most classic experimental model of polyarthritis which has been widespread used for preclinical testing of many anti-arthritic drugs (Bendele 2001). The paw edema in this model is caused by joint effusion after inflammation of synovial membrane, which can directly reflect the severity of arthritis (Bendele 2001; Hu et al. 2010). In the present study, NI could profoundly reduce forelimb swelling, but not the hind limb in AA model. It was consistent with TCM treatment on upper limb arthromyodynia. Why was NI only good at treating forelimb swelling in AA model? There may be differences in drug targets of forelimb and hind limb.

Many studies showed that P2X receptors activation had been demonstrated in *in vivo* pain models, including the rat hindpaw and knee-joint preparations, as well as in inflammatory models (Burnstock 2000; Seino et al. 2006). P2X receptors up-regulation could lead to abnormal pain responses concerned with inflammatory injuries (Xu and Huang 2002; Li et al. 2018). And selective blocking P2X_3_ receptor was effective in a series of animal models of neuropathic and chronic inflammatory pain (North 2003; Li et al. 2018). On the other hand, P2X receptors had been shown to be distributed in the different organs (Stanfa et al. 2000). P2X_1_ expressed dominantly in smooth muscle, P2X_4_ and P2X_6_ expressed predominantly in brain, P2X_7_ most heavily expressed in cells of the immune system, P2X_2_ and P2X_3_ expressed selectively in structures connected with pain signal processing (Burnstock 2000; Xu and Huang 2002). In light of receptors P2Xs' roles in the transmission signals of inflammatory pain and different distribution in organs, we therefore assessed the forelimb/hind limb distribution of P2X receptor subtypes in normal and AA rats, respectively. In normal rats, the distribution of P2X receptor subtypes was different between forelimb and hind limb. P2X_3_ and P2X_6_ receptors were found mainly in the forelimb, whereas P2X_1_, P2X_4_ and P2X_7_ receptors were found dominantly in the hind limb. In AA rats, P2X_1_, P2X_3_, P2X_4_, P2X_5_ and P2X_7_ receptors were all increased significantly, regardless of forelimb or hind limb, but P2X_3_ receptor expression was more pronounced in forelimb than that of in hind limb. Although RA was a comprehensive disease, the models in forelimb and hind limb were never the same. Our research bore this out.

As above, NI specialized in the treatment of arthromyodynia in upper limb in TCM. Consequently, we wondered whether the relationship would be reflected in NI and P2X_3_. We therefore assessed the NI effect on the P2X_3_ expression in forelimb's experiment. The results indicated that NI could profoundly reduce P2X_3_ receptor expression in forelimb's articular cavities. It suggested that NI relieved the FCA-induced RA by inhibiting upper limb's P2X_3_ receptor. If studying a topic about P2X receptor subtypes in AA, it should be discriminated between forelimb and hind limb.

## Conclusions

**Figure 8.**
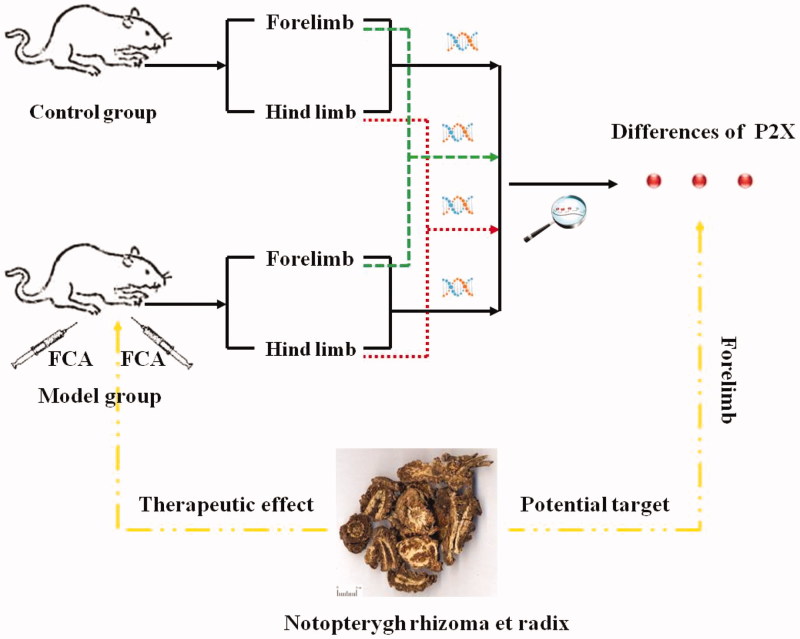
Distributive differences of P2Xs between forelimb and hind limb in rats and intervention by NI. FCA, Freund's complete adjuvant.

The scheme in Figure 8 summarizing results obtained in this study demonstrates that the distribution of P2X receptor subtypes was different in forelimb and hind limb of AA rats. P2X_3_ receptor expression was predominantly expressed in forelimb. In addition, NI relieved the FCA-induced RA by inhibiting upper limb's P2X_3_ receptor. Further research to illustrate the mechanism is in progress.
